# The Brazilian *TP53* mutation (R337H) and sarcomas

**DOI:** 10.1371/journal.pone.0227260

**Published:** 2020-01-24

**Authors:** Sahlua Miguel Volc, Cíntia Regina Niederauer Ramos, Henrique de Campos Reis Galvão, Paula Silva Felicio, Aline Silva Coelho, Gustavo Noriz Berardineli, Natalia Campacci, Cristina da Silva Sabato, Lucas Faria Abrahao-Machado, Iara Viana Vidigal Santana, Nathalia Campanella, André van Helvoort Lengert, Daniel Onofre Vidal, Rui Manuel Reis, Caio F. Dantas, Robson C. Coelho, Erica Boldrini, Sergio Vicente Serrano, Edenir Inêz Palmero

**Affiliations:** 1 Oncogenetics Department, Barretos Cancer Hospital, Barretos, São Paulo, Brazil; 2 Molecular Oncology Research Center, Barretos Cancer Hospital, Barretos, São Paulo, Brazil; 3 Center of Molecular Diagnosis, Barretos Cancer Hospital, Barretos, São Paulo, Brazil; 4 Pathology Department, Barretos Cancer Hospital, Barretos, São Paulo, Brazil; 5 Barretos Children's Cancer Hospital, Barretos, São Paulo, Brazil; 6 Life and Health Sciences Research Institute (ICVS), Health Sciences School, University of Minho, Braga, Portugal; 7 ICVS/3B's-PT Government Associate Laboratory, Braga/Guimarães, Portugal; 8 Clinical Oncology Department, Barretos Cancer Hospital, Barretos, São Paulo, Brazil; 9 Barretos School of Health Sciences, Dr. Paulo Prata-FACISB, São Paulo, Brazil; University of Texas MD Anderson Cancer Center, UNITED STATES

## Abstract

Sarcomas represent less than 1% of all solid neoplasms in adults and over 20% in children. Their etiology is unclear, but genetic susceptibility plays an important role in this scenario. Sarcoma is central in Li-Fraumeni Syndrome (LFS), a familial predisposition cancer syndrome. In Brazil, the high prevalence of p.Arg337His mutations in the *TP53* gene brings about a unique condition: a cluster of LFS. In the present work, we studied 502 sarcoma patients not selected by age or family history in an attempt to assess the impact of the so-called “Brazilian germline *TP53* mutation” (p.Arg337His) on this tumor type. We found that 8% of patients are carriers, with leiomyosarcoma being the main histologic type of sarcoma, corresponding to 52.5% of the patients with the mutated *TP53* gene. These findings emphasize the importance of genetic counseling and can better guide the management of sarcoma patients.

## Introduction

Sarcomas are malignant neoplasms of mesenchymal origin [1]. Considered rare, they comprise less than 1% of all tumors in adults [2]. In children, sarcomas account for over 20% of solid tumors [3]. The etiology of sarcoma is not well-known, but some risk factors are well described, such as the association of HHV8 and Kaposi´s sarcoma in HIV-positive individuals, previous radiation therapy, chemical exposure (such as vinyl chloride) and lymphedema [4].

One of the most studied risk factors for sarcoma is genetic susceptibility. Several different conditions may be involved, such as neurofibromatosis (*NF1*), mutations in the *RB* gene (retinoblastoma) and Li-Fraumeni syndrome (LFS, involving mutation of the *TP53* gene). Sarcoma has been known to be involved in LFS since the first description in 1969 [5] and remains in all subsequent categorizations, denoting its importance in that context [6].

In Brazil, LFS has a high prevalence, mainly due to a specific point mutation in the *TP53* gene, p.Arg337His, also known as R337H [7,8]. Since the first description of this mutation, named “the Brazilian germline *TP53* mutation” [9], researchers have been trying to establish its clinical behavior [10,11,12]. Families are heterogeneous, and despite having the same mutation, tumor patterns are quite different. Some families have adrenocortical tumors (ACT) and no other tumors, and some have many different tumor types. The background of this behavior has yet to be defined. In addition, regarding the clinical management of these R337H families, clinical surveillance protocols have been designed to organize patients’ follow-up in LFS. However, each tumor type has its own characteristics, hindering an application of these protocols in such heterogeneous families as those with “Brazilian germline *TP53* mutation” [13].

Populational ancestry may influence epidemiology, and it is an important factor to be considered in regard to pathogenic mutations. Brazil is the largest country in South America, and its population is the product of Native Americans, Europeans and Africans that intermixed over the centuries, placing Brazil as a classical model of human admixture. Recent studies have characterized Brazilian ancestry [14]; however, to the best of our knowledge, no ancestry study on Brazilian LFS patients has been conducted so far.

To clarify this issue, we evaluated the frequency of the *TP53* p.Arg337His mutation in sarcoma patients not selected by family history in a Brazilian cancer reference center. We intend to add information that contributes to the state of the art in sarcoma, a highly lethal tumor, as well as improves our understanding of frequency of the R337H mutation among sarcoma cases not selected based on clinical criteria. In addition, we also defined the ancestral substructure for all participants.

## Materials and methods

### Study subjects

A total of 701 patients treated at the Barretos Cancer Hospital between 2008 and 2016 were included in the study. Because a large proportion of the cases did not have blood samples available, frozen tumor tissue was analyzed for the purposes of this study. Samples were selected from the Institutional Biobank, macrodissected and revised by a board of pathologists who decided the best area to be analyzed (areas with tumor content higher than 60% and necrosis lower than 20%). A total of 199 cases were excluded due to different issues, mainly because of insufficient material, leading to a total of 502 sarcomas analyzed. In cases where the R337H mutation could be identified in the tumor, DNA from blood or normal tissue was analyzed when available.

Clinical and histopathological data as well as information regarding the family history of cancer were collected from the patient’s clinical chart. This study was approved by the local institutional ethical committee (approval number 866/2014). All patients provided written informed consent.

### Genotyping

Genomic DNA was extracted from peripheral blood leukocytes or tumor tissue using a DNA Blood and Tissue kit (*Qiagen*), following the manufacturer’s instructions. For the detection of *TP53* p.Arg337His, samples were PCR-amplified and analyzed by RFLP using the HhaI enzyme as described elsewhere [15]. Samples with a PCR-RFLP profile suggestive of positivity were confirmed by bidirectional Sanger sequencing. Moreover, in cases where the mutation was identified in tumor DNA, available blood or normal tissue samples were used to confirm the mutation by bidirectional Sanger sequencing.

### Ancestry analysis

A panel of 46 AIM-INDELs was performed to evaluate the population admixture proportions considering four continental origins (Africa, Europe, East Asia and Native South America) [16,17,18]. Primer sequences and PCR conditions were obtained according to Pereira et al [16]. For that purpose, a 46-multiplex PCR followed by capillary electrophoresis was performed on an ABI 3500 xL Genetic Analyzer (*Applied Biosystems*) according to the manufacturer’s instructions. The electropherograms were evaluated, and genotypes were automatically assigned with GeneMapper v4.1 (*Applied Biosystems*).

Structure v2.3.3 software [19] was used to estimate the ancestry proportions of each patient. Considering the historical formation of Brazil’s population, we assumed the four major population groups (Native Americans, Europeans, Africans and East Asians, K = 4) applied to the genetic makeup of the Brazilian population. The structure consisted of 100,000 burn-in steps followed by 100,000 Markov Chain Monte Carlo (MCMC) iterations. The option ‘Use population Information to test for migrants’ was used with the Admixture model, taking into account the allele frequencies that were correlated, and allele frequencies were updated using only individuals with POPFLAG = 1. (HGDP-CEPH samples were used as references).

### Statistical analysis

The data were described by means, standard deviations, minima and maxima for the quantitative variables and by the absolute and relative frequencies for the qualitative variables.

The relationship between the clinical characteristics and the R337H status was verified by chi-square or Fisher's exact test. The combined relationship of the variables was evaluated through multiple logistic regression, considering all the variables that were significant at a level of 20% in the univariate analysis.

A significance level of 5% was utilized, and the analyses were performed with SPSS v21.

## Results

Among the 701 cases included, 502 were analyzed. Exclusions were mostly due to insufficient material. All sarcomas were classified according to the latest WHO (World Health Organization) classifications [20] and were distributed among the patients as described in Table 1.

**Table 1:** Patients’ classification according to histologic subtypes.

Tumor subtype	Number of patients (%)
Osteosarcoma	98 (19.6)
Liposarcoma	67 (13.4)
Leiomyosarcoma	65 (12.9)
Synovial Sarcoma	55 (11.0)
Chondrosarcoma	32 (6.4)
Ewing Sarcoma	22 (4.4)
Rhabdomyosarcoma	18 (3.6)
Dermatofibrosarcoma	17 (3.4)
Myxofibrosarcoma	15 (3.0)
Undifferentiated Pleomorphic Sarcoma	15 (3.0)
Spindle Cell Sarcoma, NOS	14 (2.8)
Undifferentiated Pleomorphic Sarcoma	13 (2.6)
Angiosarcoma	10 (2.0)
Malignant Peripheral Nerve Sheath Tumor (MPNST)	7 (1.4)
Other	54 (10.5)
**Total**	**502 (100%)**

NOS: Not Otherwise Specified

In the entire group, the sex distribution was balanced: 48.5% were women and 51.5% were men. The ages ranged from 1 month to 91 years, with a mean age of 40 years (median age of 43 years). It should be emphasized that, in the hospital where the study was conducted, the majority of patients were adults; however, we analyzed 58 patients under 14 years of age.

The patients’ clinical analysis revealed advanced cancer stages (stages III and IV) in the vast majority of the group: 27.2% with stage IV (n = 132) and 40.9% (n = 199) with stage III, followed by 25.7% and 6.2% with stages II and I, respectively. When we evaluated the clinical stage in relation to age, there was no statistical significance by Cox logistic regression analysis (p = 0.030).

We found that 40 cases (8%) carried the p.Arg337His mutation. In 90% of the cases (36/40), the origin of the mutation could be verified (DNA from the blood or normal tissue was available), with all of them being germline. Comparative analysis of the sequencing profile between tumor and germline DNA suggested loss of the wild-type allele in 80% of the cases (S1 Fig). Among the four cases with only tumor available for testing, loss of the wild-type allele was observed in two cases. Table 2 provides a detailed description of the 40 mutated cases.

**Table 2:** Characteristics of patients with p.Arg337His mutations.

ID	Age at diagnosis	Mutation origin	Histologic subtype	Families' tumors (gender, age at diagnosis)	Vital status
**1**	19	germline	leiomyosarcoma	prostate leiomyosarcoma (M, 49), non-smoking lung cancer (M, 60)	alive
**2**	37	germline	myxofibrosarcoma	breast cancer (F, 60)	alive
**3**	28	germline	liposarcoma	malignant CNS (F, 28), colorectal cancer (M, 50), prostate cancer (M, 40)	alive
**4**	46	germline	leiomyosarcoma	None	alive
**5**	59	germline	leiomyosarcoma	None	dead
**6**	52	germline	myxofibrosarcoma	stomach cancer (M, 53), breast cancer (F, 51)	alive
**7**	52	germline	leiomyosarcoma	colorectal cancer (F, 60)	dead
**8**	25	germline	myxofibrosarcoma	malignant CNS (F, 45), leiomyosarcoma (M, 64), melanoma (F, 67)	dead
**9**	47	not tested	Undifferentiated Pleomorphic Sarcoma	None	dead
**10**	63	germline	leiomyosarcoma	breast cancer (F, 29; F, 37; F, 50; F, 80, F, 63), colorectal cancer (M, 58), esophagus cancer (M, 52)	alive
**11**	60	germline	Undifferentiated Pleomorphic Sarcoma	esophagus cancer (M, 70), pediatric cancer (F, 12)	dead
**12**	48	germline	Synovial sarcoma	breast cancer (F, 35), non-smoking lung cancer (F, 20), colorectal cancer (M, 30)	alive
**13**	46	germline	fibrosarcoma	head and neck cancer (F, 50)	dead
**14**	37	germline	liposarcoma	breast cancer (F, 70; F, 58), bilateral breast cancer (F, 50), sarcoma (M, 49), colorectal cancer (M, 58), urothelial cancer (F, 66), multiple tumors in one individual (F, 60; F, 58)	dead
**15**	29	germline	leiomyosarcoma	prostate cancer (M, ?; M, ?)	dead
**16**	49	germline	leiomyosarcoma	pediatric leukemia (F, 6), breast cancer (F, 30)	alive
**17**	37	not tested	leiomyosarcoma	Sarcoma (M, 34)	dead
**18**	60	germline	leiomyosarcoma	leukemia (M, 40), non-smoking lung cancer (F, 78), head and neck cancer (M, 25)	alive
**19**	68	germline	undifferentiated pleomorphic sarcoma	no information	dead
**20**	52	germline	myxoid sarcoma	lung cancer (M, 60), stomach cancer (M, 50; M, 50)	alive
**21**	35	not tested	Osteosarcoma	stomach cancer (M, ?)	dead
**22**	53	germline	Undifferentiated Pleomorphic Sarcoma	non-smoking lung cancer (M, 18), breast cancer (F, 44; F, 50; F, 50), colorectal cancer (F, 84), pancreas (F, ?)	dead
**23**	50	germline	leiomyosarcoma	leukemia (F, 1), hepatocellular carcinoma (M, 37)	alive
**24**	50	germline	leiomyosarcoma	malignant CNS (F, 28), adrenal cancer (F, 31), non-smoking lung cancer (F, 45), stomach cancer (M, 60)	Alive
**25**	66	germline	spindle cell sarcoma, NOS	malignant CNS (M, 60; F, 42; M, 41), breast cancer (F, 50; F, 40), bilateral breast cancer (F, 32), leukemia (M, 12), lung cancer (M, 50), stomach cancer (F, 45; M, 45), multiple tumors in one individual (F, 32), melanoma (F, 44)	Alive
**26**	50	germline	leiomyosarcoma	colorectal cancer (W, 70)	Alive
**27**	53	germline	leiomyosarcoma	sarcoma (F, 68), adrenal tumor (M, 23), non-smoking lung cancer (M, 44; F, 73; F, 49; F, 54), stomach cancer (M, 61), leukemia (F, 40), renal cancer (F, 39), GIST (M, 59)	Alive
**28**	49	germline	Malignant Peripheral Nerve Sheath Tumor	adrenal cancer (F, 1; F, 32), colorectal cancer (F, 35), stomach cancer (F, 55; F, ?), lung cancer (M, 55)	Alive
**29**	61	germline	leiomyosarcoma	malignant CNS (F, 62), lung cancer (M, 65), sarcoma (F, 76), colorectal cancer (F, 40)	Dead
**30**	75	germline	liposarcoma	None	Alive
**31**	60	germline	leiomyosarcoma	adrenal cancer (M, 5), sarcoma (F, 60)	Alive
**32**	18	germline	osteosarcoma	no information	Dead
**33**	64	germline	leiomyosarcoma	adrenal cancer (F, 2), colorectal cancer (F, 45), leukemia (F, 62)	Dead
**34**	58	germline	leiomyosarcoma	None	Alive
**35**	59	germline	leiomyosarcoma	prostate cancer (M, 83; M, 75), colorectal cancer (F, 50)	Alive
**36**	61	germline	leiomyosarcoma	breast cancer (F, 64; F, 50; F, 40; F, 50), non-smoking lung cancer (F, 80)	Alive
**37**	42	germline	leiomyosarcoma	no information	Alive
**38**	41	germline	rhabdomyosarcoma	no information	Dead
**39**	82	germline	spindle cell sarcoma, NOS	no information	Dead
**40**	21	not tested	leiomyosarcoma	colorectal cancer (F, 60)	Alive

CNS: central nervous system; GIST: gastrointestinal stromal tumor; NOS: not otherwise specified; F: female; M: male.

**Fig 1:**
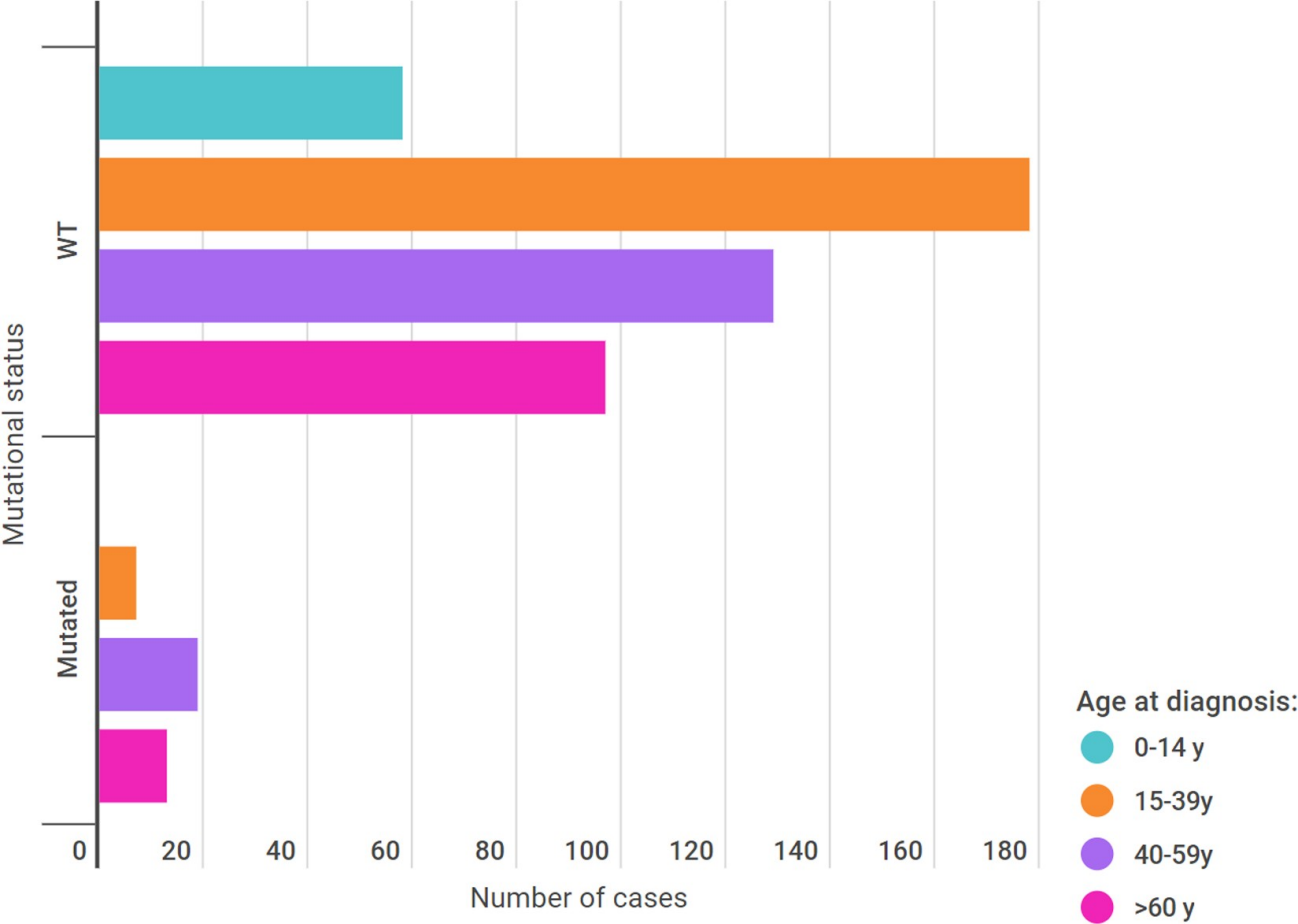
Age distribution according to mutational status.

When patients were stratified according to the mutational status, it was found that adulthood is the principal age group for mutation, considering those of 40 years of age or more (Fig 1). Although pediatric patients constituted 11.6% of the cohort, we did not find any R337H-positive cases in this group. The youngest patient with the *TP53* mutation was 18 years old, and the oldest was 84 years old. The age distribution according to mutational status is shown in Fig 1.

Information regarding cancer history in families positive for the mutation was available for 87.5% of the cases. Among those, 85.7% reported a positive family history, as shown in Table 2. In 83% of the families, there were tumors that constituted part of the LFS spectrum, although the R337H status was not verified in the relatives.

**Fig 2:**
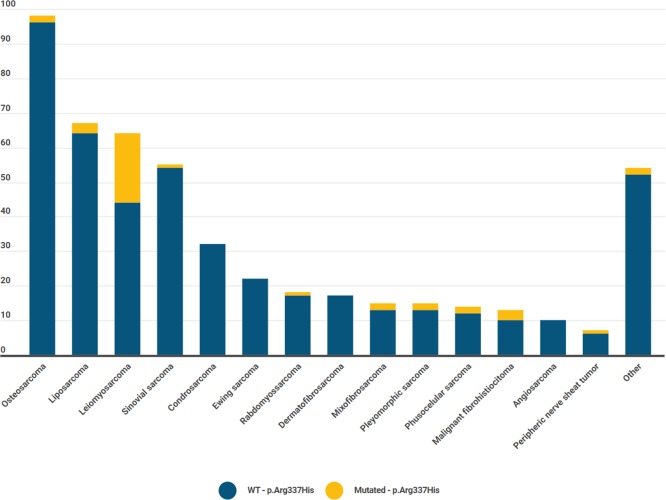
Mutational status according to sarcoma subtype.

When analyzing tumor types in mutated cases, leiomyosarcoma was the predominant subtype (Fig 2).

Among the 40 patients positive for the *TP53* mutation, 8 presented with more than one tumor. In almost all patients, sarcoma was the second tumor diagnosed (Table 3).

**Table 3:** Patients with multiple tumors.

Patient	Sex	First tumor (age at diagnosis)	Second tumor (age at diagnosis)
**2**	**F**	Phyllodes breast Tumor (36)	Myxofibrosarcoma (37)
**4**	**F**	Renal cancer (38)	Leiomyosarcoma (46)
**10**	**M**	Thyroid tumor (58)	Leiomyosarcoma (63)
**18**	**F**	Breast cancer (49)	Leiomyosarcoma (60)
**20**	**M**	Prostate cancer (42)	Myxoid sarcoma (52)
**26**	**F**	Breast cancer (42)	Leiomyosarcoma (50)
**35**	**F**	Thyroid cancer (61)	Leiomyosarcoma (59)
**36**	**F**	Leiomyosarcoma (61)	Breast cancer (68)

F = female; M = male

**Fig 3:**
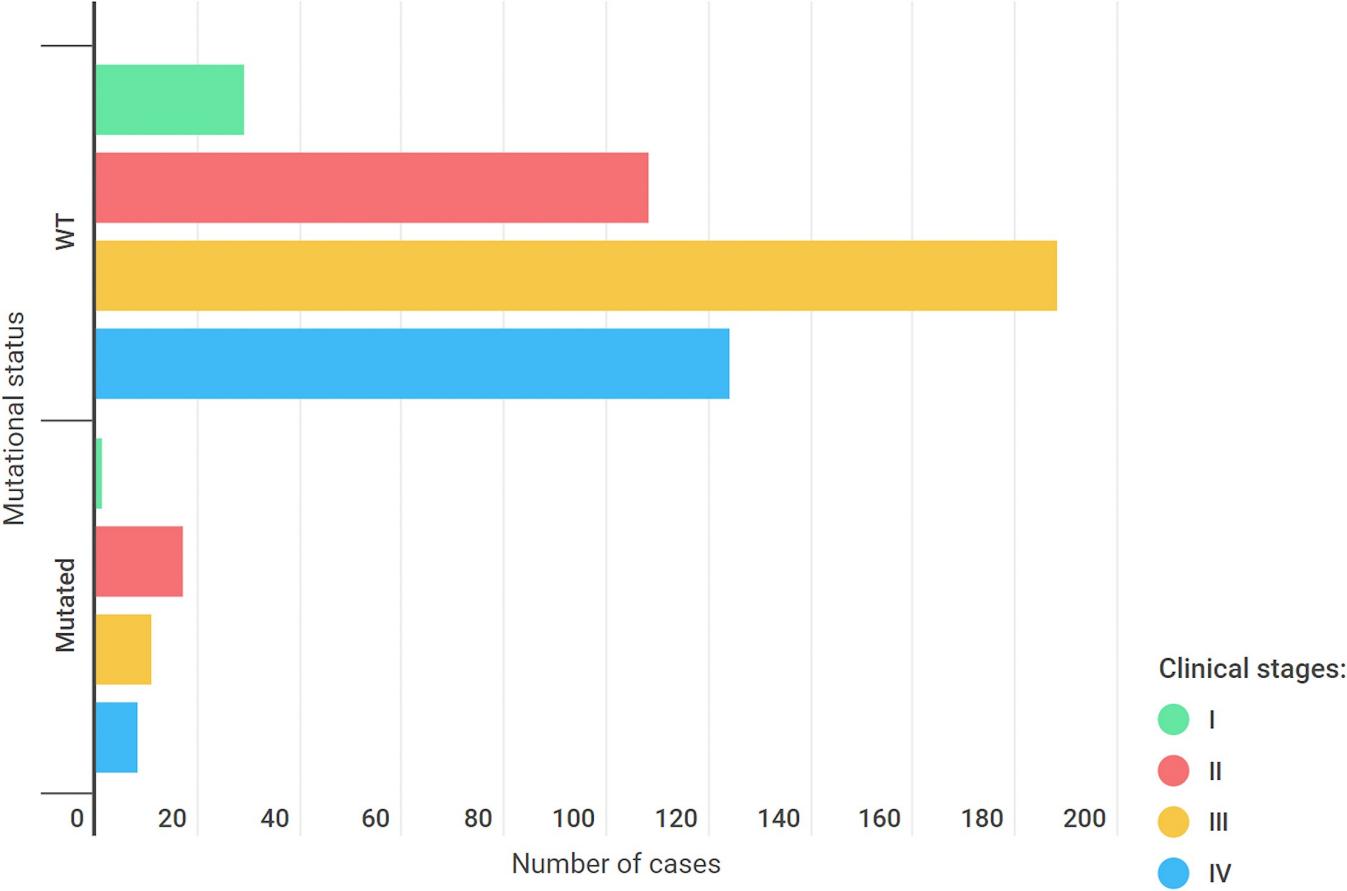
Clinical stages among wild-type and mutated cases.

The vast majority of our cohort (68%) had advanced clinical stages (III and IV) of cancer. There was no statistically significant difference when comparing the clinical stages between mutated and wild-type cases (p = 0.30) (Fig 3).

**Fig 4:**
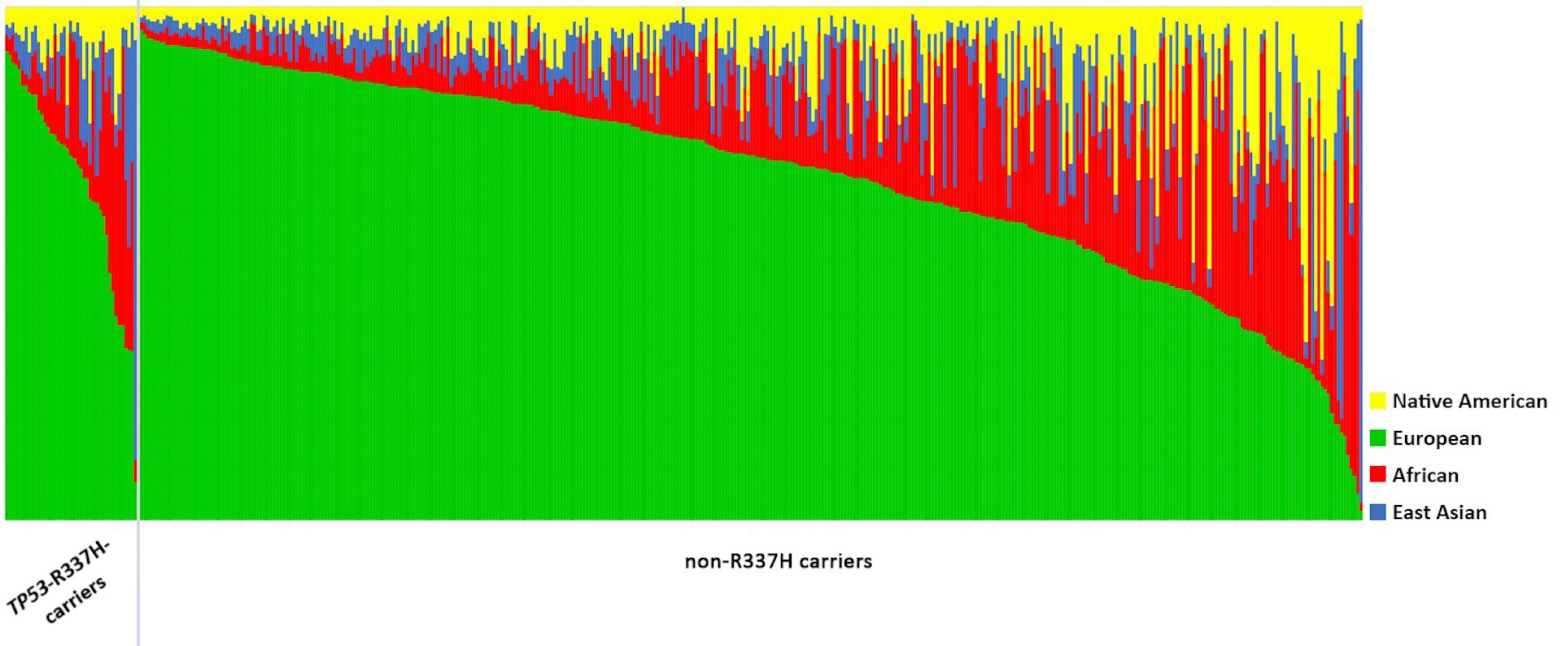
Ancestral profile of all patients analyzed. Left: Patients with the R337H mutation. Right: Patients with the WT phenotype.

Considering the declared ethnicity, 60% of the patients from our cohort were white. Ancestry analysis was performed for 411 patients, and the results are compatible with the declared data, reporting that the median value for European ancestry was 67.1%, followed by African (15.9%), East Asian (7.2%) and Native American (9.8%) (Fig 4). Among the mutated cases, a total of 39 patients with the Brazilian founder pathogenic variant in *TP53* (c.1010G>A; p.Arg337His) were analyzed (Fig 4). For these 39 individuals, the average ancestry proportions were 66.3% for European, followed by African (15.6%), East Asian (10.7%) and Native American (7.4%). Using the Mann-Whitney test, we did not find any correlation between the ancestral profile and the presence/absence of the pathogenic variant in *TP53* (p.Arg337His).

**Fig 5:**
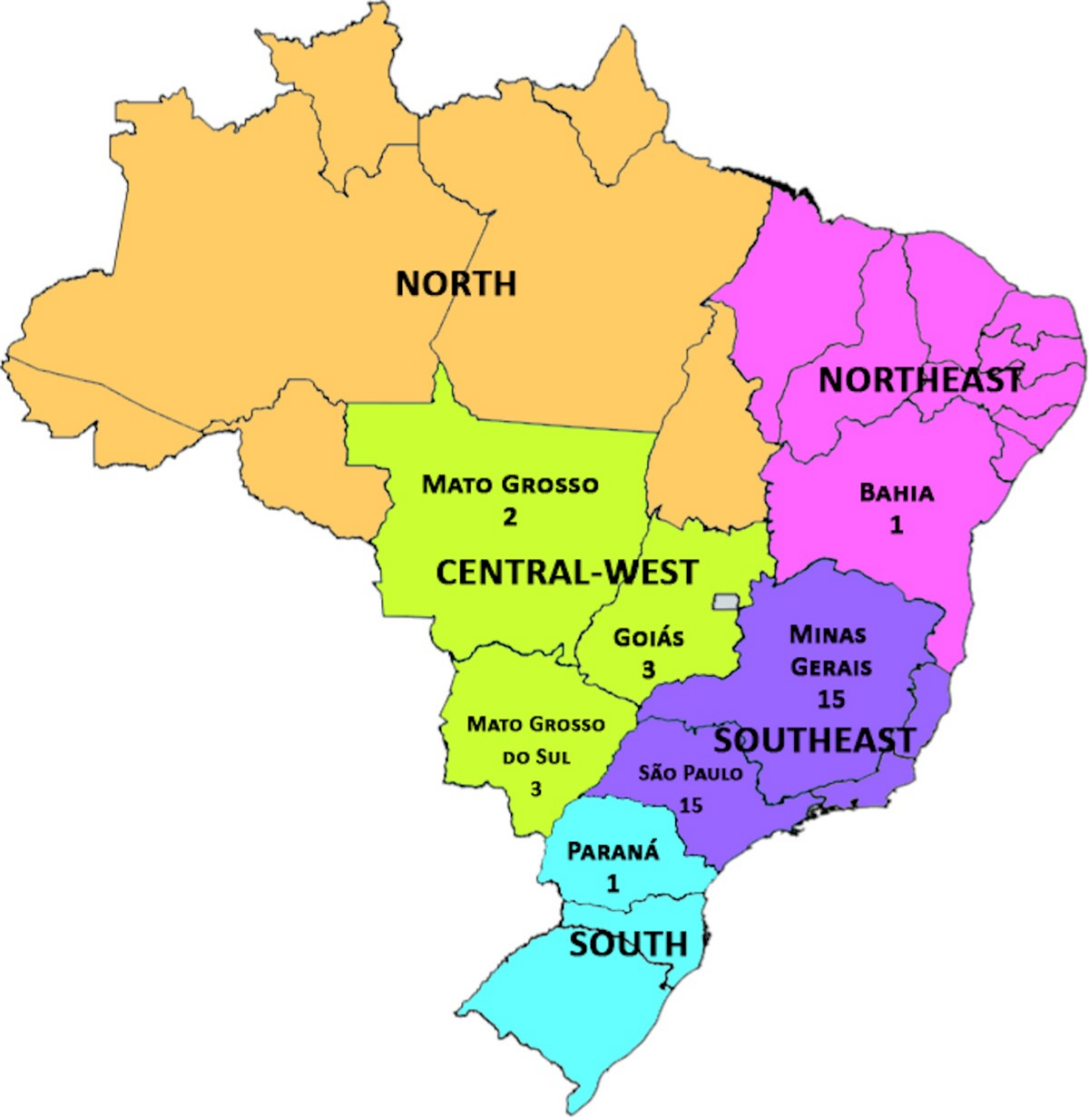
Brazilian map depicting the birthplace of patients with the R337H mutation (numbers in the map describe the number of patients with the R337H mutation who came from that specific state).

It is known that the “Brazilian germline *TP53* mutation” occurs predominantly in southern Brazil [7,8,10,15,21]. However, the institution where this study took place receives patients from outside of southern Brazil. Therefore, our study could identify mutations in the northern and central-western regions, as depicted in Fig 5.

## Discussion

Since its first description, the “Brazilian germline *TP53* mutation” has been an intriguing point of discussion in oncogenetics. Garritano and cols. proved a founder effect for this particular mutation [22]. It has a preferential geographical localization and a peculiar clinical behavior. This mutation was first described in southern Brazil [9]. Our data demonstrate that the mutation is no longer geographically restricted, as some of our cases are from the central-west and northern regions, emphasizing the national importance of this condition. This spread reflects the internal migration within Brazil and has a direct impact on public health care since the mutation predisposes carriers to multiple cancer types.

We found that 8% of a nonselected, random sarcoma population are carriers of the R337H –*TP53* pathogenic variant. Sarcoma is the lead tumor type in the original description of LFS and appeared in our study as an important issue. Previous Brazilian studies have found an unexpectedly high prevalence of this mutation in other types of LFS tumors, for instance, adrenocortical and plexus choroid carcinoma [10].

A review of the IARC *TP53* database analyzed the types of mutations in and ages of sarcoma patients and demonstrated an age-dependent variation in these patients. They concluded that for *TP53* mutation carriers, 67% of sarcomas appeared before 20 years of age [23]. However, our data demonstrate quite different behavior, with 95% of the sarcomas being diagnosed after the age of 20 and 77.5% after 40 years of age. A possible explanation for these differences comes from our institution´s characteristics, where the Clinical Oncology service is bigger and older than the Pediatric Oncology service. However, we analyzed 58 sarcoma patients under 14 years of age, which is a robust number compared to those of previous Brazilian studies [10].

Regarding histopathology, following the latest WHO sarcoma classification, our data show a regular distribution pattern [1]. When only patients with the R337H mutation were analyzed, a particular subtype, leiomyosarcoma, was revealed, accounting for 52.5% of the sarcomas. According to the IARC *TP53* database, leiomyosarcoma corresponds to 9.1% of the sarcomas in *TP53* mutation carriers, showing once again the particular behavior of the p.Arg337His pathogenic variant [23].

It is important to highlight that leiomyosarcomas have a poor prognosis. They do not respond well to chemotherapy and radiotherapy [24]. If resected, cure might be possible, but if this approach is not possible, mortality could be high. Therefore, early diagnosis is crucial for a better outcome.

Family history is an important indicator of hereditary cancer identification. In our registry, 75% of the patients with the *TP53* mutation had family members with cancer (85.7% of those whose information regarding cancer history in families was available). Among the cancer subtypes in relatives, some were more frequent, such as lung cancer, breast cancer, malignant central nervous system tumor, colorectal and stomach cancer, leukemia and adrenal tumor. However, in approximately 15% of the mutated cases, there was no cancer family history. This emphasizes that, in Brazil, leiomyosarcoma should be considered a core LFS tumor and that the mutation should be accessed even in the absence of cancer in family history. Such a diagnosis might benefit these patients, offering specific preventive protocols to avoid second tumors.

Some positive patients had multiple malignant tumors, which could suggest details to improve surveillance protocols. Since sarcomas appeared as a second tumor in 7 of 8 patients with multiple tumors in our data, cancer patients who carried the p.Arg337H mutation should continue to be monitored with clinical cancer surveillance protocols.

Ancestry was also analyzed, and no correlation with the mutation could be demonstrated. The declared ethnicity and the molecular ancestry profile analysis were similar, and no significant differences were found. Brazil was colonized by different ethnicities, resulting in a highly mixed population, and we found this phenomenon in our study population as well.

In terms of the limitations of this study, it is important to note that, although our goal was to analyze a germline mutation, due to lack of availability of normal samples (blood/normal tissue), tumor samples were evaluated instead. However, all mutated cases with germline material available had the alteration confirmed (36/40 cases), and in all cases, the germline origin of the alteration was confirmed. In addition, somatic R337H mutations in the IARC TP53 database (R19) occurred in 4 tumors among 29895 investigated (0.013%). These findings allow us to infer that the mutation was also germline in the remaining cases where germline DNA was unavailable for analysis.

In conclusion, we highlight that our data contribute to a better understanding of the “Brazilian germline *TP53* mutation” and its clinical behavior, which can help to improve surveillance protocols.

## Supporting information

S1 Fig Comparative analysis of the sequencing profile between tumor and germline DNA. (TIFF)
