# Supplementary figures and images for: The Brazilian TP53 mutation (R337H) and sarcomas
Source: PLoS One. 2020 Jan 24;15(1):e0227260. doi: 10.1371/journal.pone.0227260 (PMC6980636; doi:10.1371/journal.pone.0227260)

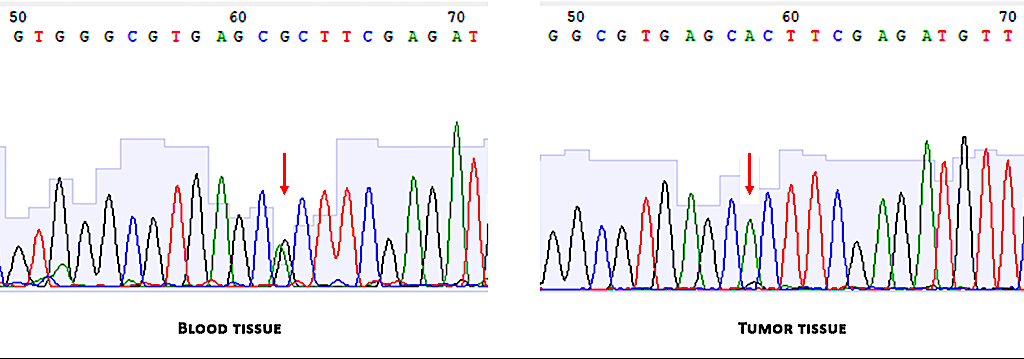

Supplement: S1 Fig — (TIFF) [file pone.0227260.s001.tiff]
